# Migrasomes trigger innate immune activation and mediate transmission of senescence signals across human cells

**DOI:** 10.1093/lifemedi/lnad050

**Published:** 2023-12-08

**Authors:** Xiaoqian Liu, Haifeng Jiao, Baohu Zhang, Sheng Zhang, Kaowen Yan, Jing Qu, Weiqi Zhang, Li Yu, Guang-Hui Liu

**Affiliations:** State Key Laboratory of Stem Cell and Reproductive Biology, Institute of Zoology, Chinese Academy of Sciences, Beijing 100101, China; Savaid Medical School, University of Chinese Academy of Sciences, Beijing 100049, China; Institute for Stem Cell and Regeneration, Chinese Academy of Sciences, Beijing 100101, China; Beijing Institute for Stem Cell and Regenerative Medicine, Chinese Academy of Sciences, Beijing 100101, China; State Key Laboratory of Membrane Biology, Tsinghua University-Peking University Joint Center for Life Sciences, Beijing Frontier Research Center for Biological Structure, School of Life Sciences, Tsinghua University, Beijing 100084, China; State Key Laboratory of Stem Cell and Reproductive Biology, Institute of Zoology, Chinese Academy of Sciences, Beijing 100101, China; Savaid Medical School, University of Chinese Academy of Sciences, Beijing 100049, China; Savaid Medical School, University of Chinese Academy of Sciences, Beijing 100049, China; State Key Laboratory of Membrane Biology, Institute of Zoology, Chinese Academy of Sciences, Beijing 100101, China; Savaid Medical School, University of Chinese Academy of Sciences, Beijing 100049, China; Institute for Stem Cell and Regeneration, Chinese Academy of Sciences, Beijing 100101, China; Beijing Institute for Stem Cell and Regenerative Medicine, Chinese Academy of Sciences, Beijing 100101, China; State Key Laboratory of Membrane Biology, Institute of Zoology, Chinese Academy of Sciences, Beijing 100101, China; State Key Laboratory of Stem Cell and Reproductive Biology, Institute of Zoology, Chinese Academy of Sciences, Beijing 100101, China; Savaid Medical School, University of Chinese Academy of Sciences, Beijing 100049, China; Institute for Stem Cell and Regeneration, Chinese Academy of Sciences, Beijing 100101, China; Beijing Institute for Stem Cell and Regenerative Medicine, Chinese Academy of Sciences, Beijing 100101, China; Aging Biomarker Consortium, Beijing 100101, China; Savaid Medical School, University of Chinese Academy of Sciences, Beijing 100049, China; Institute for Stem Cell and Regeneration, Chinese Academy of Sciences, Beijing 100101, China; Aging Biomarker Consortium, Beijing 100101, China; CAS Key Laboratory of Genomic and Precision Medicine, Beijing Institute of Genomics, Chinese Academy of Sciences and China National Center for Bioinformation, Beijing 100101, China; State Key Laboratory of Membrane Biology, Tsinghua University-Peking University Joint Center for Life Sciences, Beijing Frontier Research Center for Biological Structure, School of Life Sciences, Tsinghua University, Beijing 100084, China; Savaid Medical School, University of Chinese Academy of Sciences, Beijing 100049, China; Institute for Stem Cell and Regeneration, Chinese Academy of Sciences, Beijing 100101, China; Beijing Institute for Stem Cell and Regenerative Medicine, Chinese Academy of Sciences, Beijing 100101, China; State Key Laboratory of Membrane Biology, Institute of Zoology, Chinese Academy of Sciences, Beijing 100101, China; Aging Biomarker Consortium, Beijing 100101, China; Advanced Innovation Center for Human Brain Protection, and National Clinical Research Center for Geriatric Disorders, Xuanwu Hospital Capital Medical University, Beijing 100053, China

**Keywords:** migrasomes, cellular senescence, endogenous retrovirus, senescence-associated secretory phenotype, aging

## Abstract

Aging is a complex and heterogeneous process, raising important questions about how aging is differently impacted by underlying genetics and external factors. Recently, migrasomes, newly discovered organelles, have been identified to play important roles in various physiological and pathological processes by facilitating cell-to-cell communication. Thus far, their involvement in cellular senescence and aging remains largely unexplored. In this study, we aimed to investigate how migrasomes impact on cellular aging by leveraging multiple cellular senescence models, including replicatively senescent (RS), pathologically senescent and stress-induced senescent human mesenchymal stem cells (hMSCs), as well as RS human primary fibroblasts. In all cellular aging models, we detected an enhanced formation of migrasomes. Notably, migrasomes in senescent cells exhibited an accumulation of numerous aging hallmarks, such as dysfunctional mitochondria, endogenous retroviruses, and senescence-associated pro-inflammatory cytokines. Furthermore, we discovered that migrasomes derived from senescent cells can be taken up by young cells, thereby transferring aging signals and subsequently causing premature senescence phenotypes in recipient cells. Mechanistically, we found that treatment with migrasomes derived from senescent cells activated the innate immune response. Thus, our study sheds light on a pivotal role of migrasomes in mediating the contagiousness of aging.

## Introduction

Aging, a major risk factor of many chronic diseases, is accompanied by functional decline in multiple tissues and characterized with twelve hallmarks, including cellular senescence, senescence-associated secretory phenotype (SASP), and mitochondrial dysfunction [1–5]. Recently, altered cell–cell communication was identified as a new hallmark of aging. Our recent discovery revealed that resurrection of human endogenous retroviruses (HERVs), remnants of ancient viral infections, which are present in the human genome, act as a novel biomarker and driver of aging [6–8]. We found that senescent cells reactivate and release HERVs, which are then transmitted into young cells in a paracrine manner, leading to the senescence of recipient cells [9, 10]. Hence, our study provides compelling evidence in support of contagiousness being a feature of aging [11], a notion that requires further validation in the field of aging research. However, how HERVs and other senescence-associated mediators transmit aging information from senescent cells to non-senescent cells, thereby facilitating cell-cell communication and/or magnifying pro-aging signals, remains largely unknown.

Migrasomes, recently discovered pomegranate-like vesicle organelles with a diameter ranging from 0.5 to 3 μm, have been detected in various types of migrating cells [12–14]. They originate from local swellings on retraction fibers, become stabilized by tetraspanin (TSPAN)-based membrane domains, and are released into the medium following the breakdown of retraction fibers [15, 16]. Subsequently, surrounding neighbor cells take them up through a process referred to as migracytosis [17]. A variety of cytosolic contents are enriched in migrasomes, including cytokines, chemokines, growth factors, mRNA, proteins, and damaged mitochondria [18, 19]. Migrasomes are recognized for their critical roles in facilitating cell-cell communication and maintaining cellular homeostasis through lateral transfer or disposal of damaged cellular materials, thereby involving in physiological processes including embryonic development, and pathological processes such as tumorigenesis and cerebral amyloid angiopathy [20–25]. However, whether migrasomes serve as subcellular structural carriers for pro-senescent signaling, and play a role in the context of cellular senescence, has not been investigated.

Here, we found an increased presence of migrasomes in multiple senescent cell models. Beyond the conventional indicators of aging, such as SASP and mitochondrial fragments, migrasomes originating from senescent cells showed a notable enrichment of endogenous retroviruses. More importantly, these migrasomes have the capacity to be internalized by young cells, subsequently inducing their senescence. These findings underscore the plausible functions of migrasomes in facilitating the transmission and augmentation of pro-aging signaling.

## Results

### Senescent cells exhibited increased formation of migrasomes

**Figure 1.**
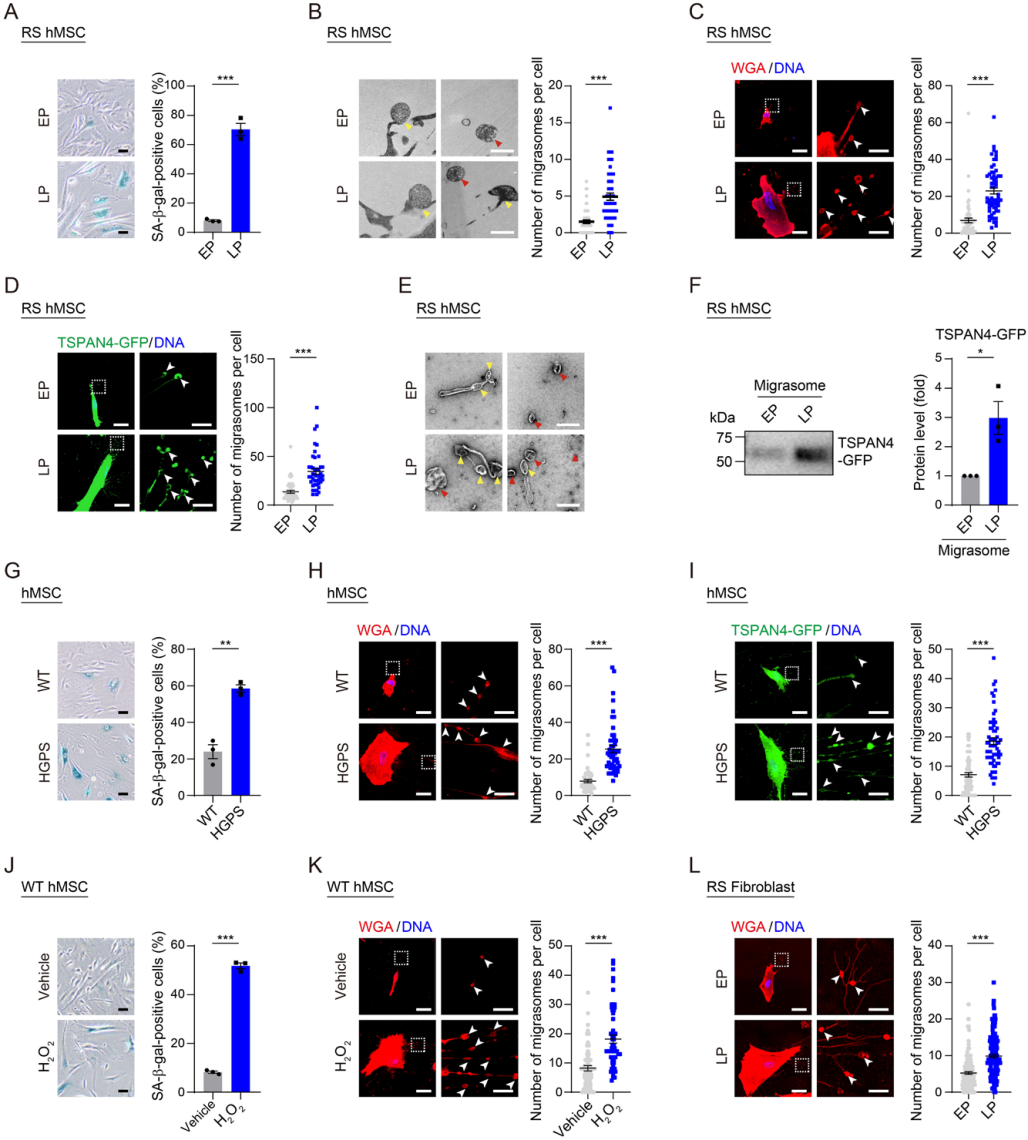
**The number of migrasomes increased during cellular senescence.**(A) SA-β-gal staining of WT hMSCs at EP (P4) and LP (P14). Left: Representative images. Scale bars, 20 μm. Right: Quantification of the percentages of SA-β-gal-positive cells. Data are presented as the mean ± SEM. *n* = 3 biological replicates. Over 100 cells were quantified in each replicate. ***P < 0.001 (*t* test). (B) TEM analysis of migrasomes in WT hMSCs at EP (P4) and LP (P14). Left: Representative images. Yellow and red arrows indicate migrasomes associated with retraction fibers or released into the extracellular environment, respectively. Scale bars, 1 μm. Right: Quantification of the number of migrasomes per cell. More than 50 cells were quantified in each group. ***P < 0.001 (*t* test). (C) Fluorescence microscopy analysis of migrasomes labeled with Alexa Fluor™ 555-conjugated WGA probe in WT hMSCs at EP (P4) and LP (P14). Left: Representative images. Scale bars, 20 μm and 4 μm (zoomed-in images). Right: Quantification of the number of migrasomes per cell. *n* = 150 cells. ***P < 0.001 (*t* test). (D) Fluorescence microscopy detection of WT hMSCs at EP (P4) and LP (P14) transduced with lentiviral vectors carrying TSPAN4-GFP. Left: Representative images. Scale bars, 20 μm and 4 μm (zoomed-in images). Right: Quantification of the number of migrasomes per cell. *n* = 50 cells. ***P < 0.001 (*t* test). (E) Negative-staining TEM analysis of purified migrasomes from EP (P4) and LP (P14) WT hMSCs transduced with lentiviral vectors carrying TSPAN4-GFP. Yellow and red arrows indicate migrasomes associated with retraction fibers or released into the extracellular environment, respectively. Scale bars, 2 μm. (F) Western blotting of TSPAN4-GFP in purified migrasomes from WT hMSCs at EP (P4) and LP (P14) transduced with lentiviral vectors carrying TSPAN4-GFP. Left: Representative images. The loading was adjusted with the same amount of protein lysates. Right: Quantification of the relative protein level of TSPAN4-GFP. Data are presented as the mean ± SEM. *n* = 3 independent experiments. *P < 0.05 (*t* test). (G) SA-β-gal staining of WT and HGPS hMSCs at P7. Left: Representative images. Scale bars, 20 μm. Right: Quantification of the percentages of SA-β-gal-positive cells. Data are presented as the mean ± SEM. *n* = 3 biological replicates. Over 100 cells were quantified in each replicate. **P < 0.01 (*t* test). (H) Fluorescence microscopy analysis of migrasomes labeled with Alexa Fluor™ 555-conjugated WGA probe in WT and HGPS hMSCs at P7. Left: Representative images. Scale bars, 20 μm and 4 μm (zoomed-in images). Right: Quantification of the number of migrasomes per cell. *n* = 150 cells. ***P < 0.001 (*t* test). (I) Fluorescence microscopy detection of WT and HGPS hMSCs at P7 transduced with lentiviral vectors carrying TSPAN4-GFP. Left: Representative images. Scale bars, 20 μm and 4 μm (zoomed-in images). Right: Quantification of the number of migrasomes per cell. *n* = 50 cells. ***P < 0.001 (*t* test). (J) SA-β-gal staining of WT hMSCs at P8 treated with H_2_O_2_. Left: Representative images. Scale bars, 20 μm. Right: Quantification of the percentages of SA-β-gal-positive cells. Data are presented as the mean ± SEM. *n* = 3 biological replicates. Over 100 cells were quantified in each replicate. ***P < 0.001 (*t* test). (K, L) Fluorescence microscopy analysis of migrasomes labeled with Alexa Fluor™ 555-conjugated WGA probe in WT hMSCs at P8 treated with H_2_O_2_ (K) or in human fibroblasts at EP (P12) and LP (P22) (L). Left: Representative images. Scale bars, 20 μm and 4 μm (zoomed-in images). Right: Quantification of the number of migrasomes per cell. *n* = 150 cells. ***P < 0.001 (*t* test).

To investigate the formation and changes of migrasomes associated with cellular senescence, we leveraged transmission electron microscopy (TEM) to visualize migrasomes in replicatively senescent (RS) human mesenchymal stem cells (hMSCs), a state in which cells have made a transition into cell cycle arrest after serial replication rounds (Fig. 1A and Supplementary Fig. S1A) [26, 27]. Within the extracellular environment of late passage (LP) wildtype (WT) hMSCs, we observed migrasome-like formations, characterized by oval-shaped membranous structures [12, 28], whereas these were barely present in the extracellular space of early passage (EP) hMSCs (Fig. 1B). In some migrasomes, we detected numerous smaller vesicles or cytosolic organelles with a pomegranate-like structure whereas others were empty or connected to retraction fibers (Fig. 1B). To analyze their formation in senescent hMSCs, we labeled migrasomes with the fluorochrome-conjugated Wheat Germ Agglutinin (WGA) probe [20, 29], and detected migrasomes positioned at branching points of retraction fibers, at the terminal extensions of these fibers, and spanning across retraction fibers (Fig. 1C). We also detected migrasomes that had become detached and released into the extracellular environment (Fig. 1C). Relative to EP hMSCs, we found that the total number of migrasomes was higher in LP hMSCs (Fig. 1C). In addition, compared to EP hMSCs, the increased volume of migrasomes and more migrasomes released into the extracellular environment were observed in LP hMSCs (Supplementary Fig. S1B). Furthermore, when transduced EP and LP hMSCs with lentiviral vectors expressing TSPAN4-GFP, a transmembrane protein and classical marker of migrasomes [12], we found again that the number of migrasomes was higher in senescent LP hMSCs relative to young EP hMSCs (Fig. 1D). Utilizing live-cell imaging, we observed a dynamic formation and movement of migarsomes in both EP and LP hMSCs (Supplementary Fig. S1C, Supplementary Movies S1 and S2). Finally, we took a biochemical approach and purified migrasomes from both EP and LP hMSCs transduced with lentiviral TSPAN4-GFP using ultracentrifugation [14, 19]. Through negative-staining TEM and fluorescence microscopy analysis, we confirmed that we had purified migrasomes with the typical vesicular structure (Fig. 1E and Supplementary Fig. S1D). Western blotting verified that elevated levels of TSPAN4-GFP were present in migrasomes derived from LP hMSCs relative to those derived from EP hMSCs (Fig. 1F). Collectively, these findings indicate that a higher number of migrasomes are formed in RS hMSCs.

Next, we sought to investigate migrasome formation in other senescent hMSC models and turned to the Hutchinson-Gilford progeria syndrome (HGPS) model, in which premature aging characteristics are caused by a dominant-negative mutation in *LMNA* (c.1824 C > T; p.G608G) (Fig. 1G and Supplementary Fig. S1E) [30–32]. Using TSPAN4-GFP and a fluorochrome-conjugated WGA probe to label migrasomes, we confirmed that the deposition of migrasomes in prematurely senescent HGPS hMSCs was increased (Fig. 1H and I). In WT hMSCs, we used hydrogen peroxide (H_2_O_2_) treatment to induce a stress-associated senescent phenotype [33–35], which also resulted in an amplified formation of migrasomes (Fig. 1J and K and Supplementary Fig. S1F). Additionally, we also detected an increased number of migrasomes in RS human fibroblasts (Fig. 1L, Supplementary Fig. S1G and H). Taken together, these data provide evidence for an augmented formation of migrasomes during cellular senescence, which can serve as a novel biomarker of aging.

### Accumulation of mitochondria was observed within migrasomes during cellular senescence

**Figure 2.**
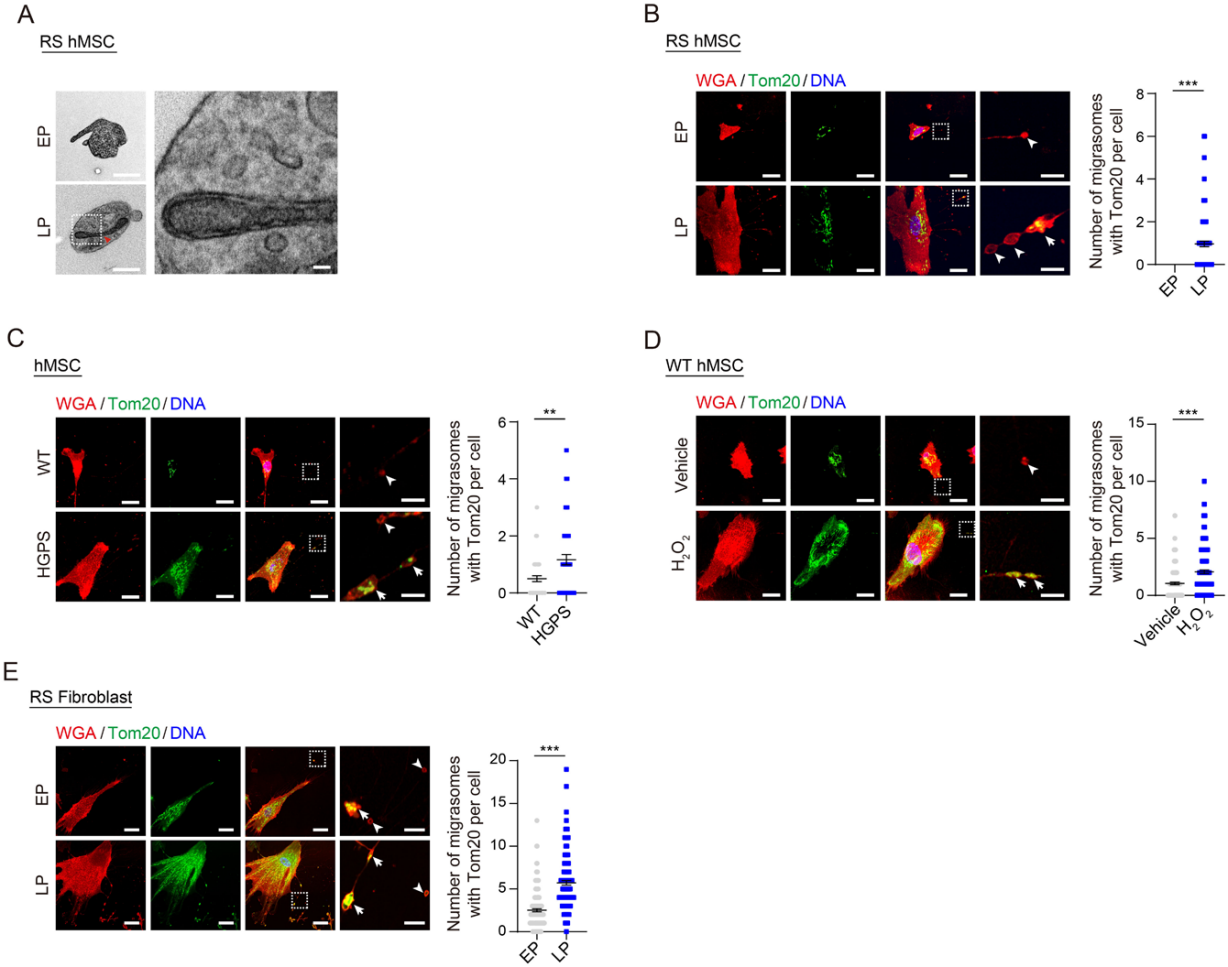
**Mitochondria were enriched within migrasomes in senescent cells.** (A) TEM analysis of mitochondria within migrasomes in WT hMSCs at EP (P4) and LP (P14). Red arrow indicates the mitochondria within migrasomes released into the extracellular environment. Scale bars, 1 μm and 100 nm (zoomed-in images). (B–E) Immunofluorescence co-staining of Tom20 with WGA probe in WT hMSCs at EP (P4) and LP (P14) (B), in WT and HGPS hMSCs at P7 (C), in WT hMSCs at P8 treated with H_2_O_2_ (D), or in human fibroblasts at EP (P12) and LP (P22) (E). Left: Representative images. Tailless and trailed arrows indicate migrasomes without or with Tom20, respectively. Scale bars, 20 μm and 4 μm (zoomed-in images). Right: Quantification of the number of migrasomes with Tom20 per cell. *n* = 150 cells. **P < 0.01, ***P < 0.001 (*t* test).

Given the heightened migrasome production in senescent cells, we were curious to investigate the contents within these migrasomes. Considering the known function of migrasomes in the deposition of damaged mitochondria [36], and the important role of mitochondrial dysfunction in aging [37–40], we first asked whether migrasomes derived from senescent cells contained increased numbers of mitochondria. With TEM analysis, we detected mitochondria within migrasomes (Fig. 2A), and upon co-staining the mitochondrial membrane protein Tom20 [41] with the WGA probe, we observed an increased abundance of mitochondria in migrasomes from LP hMSCs when compared to EP hMSCs (Fig. 2B). Consistently, we observed a parallel phenomenon in other senescent cell models, encompassing pathological progeria, stress-induced prematurely senescent hMSCs, and RS human fibroblasts (Figs. 2C–E). Taken together, these investigations reveal that mitochondria accumulate within migrasomes in senescence cells, suggesting a potential role for migrasomes in facilitating the disposal of mitochondria during cellular senescence.

### Senescent cells exhibited increased enrichment of endogenous retroviruses

**Figure 3.**
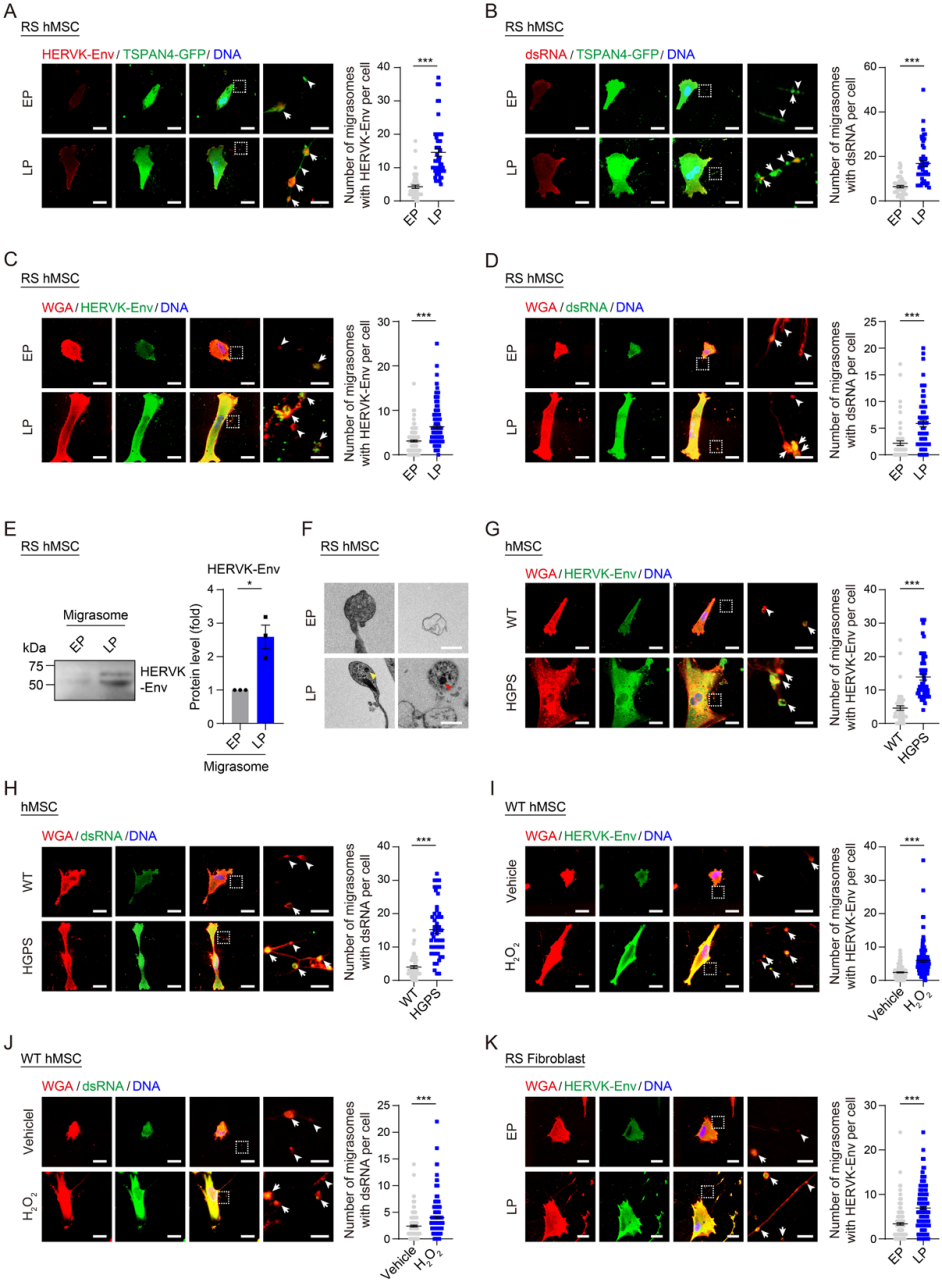
**Endogenous retroviruses accumulated in migrasomes during cellular senescence.** (A, B) Immunofluorescence staining of HERVK-Env (A) and dsRNA (B) in WT hMSCs at EP (P4) and LP (P14) transduced with lentiviral vectors carrying TSPAN4-GFP. Left: Representative images. Tailless and trailed arrows indicate migrasomes without or with HERVK-Env (A) or dsRNA (B), respectively. Scale bars, 20 μm and 4 μm (zoomed-in images). Right: Quantification of the number of migrasomes with HERVK-Env (A) or dsRNA (B) per cell. *n* = 50 cells. *** *P* < 0.001 (*t* test). (C, D) Immunofluorescence co-staining of HERVK-Env (C), or dsRNA (D) with WGA probe in WT hMSCs at EP (P4) and LP (P14). Left: Representative images. Tailless and trailed arrows indicate migrasomes composed migrasomes without or with HERVK-Env (C) or dsRNA (D), respectively. Scale bars, 20 μm and 4 μm (zoomed-in images). Right: Quantification of the number of migrasomes with HERVK-Env (C) or dsRNA (D) per cell. *n* = 150 cells. ***P < 0.001 (*t* test). (E) Western blotting of HERVK-Env in purified migrasomes from WT hMSCs at EP (P4) and LP (P14). Left: Representative images. The loading was adjusted with the same amount of protein lysates. Right: Quantification of the relative protein level of HERVK-Env. Data are presented as the mean ± SEM. *n* = 3 independent experiments. *P < 0.05 (*t* test). (F) TEM analysis of RVKPs within migrasomes in WT hMSCs at EP (P4) and LP (P14). Yellow and red arrows indicate RVKPs within migrasomes associated with retraction fibers or released into the extracellular environment, respectively. Scale bars, 1 μm. (G, H) Immunofluorescence co-staining of HERVK-Env (G) or dsRNA (H) with probe WGA probe in WT and HGPS hMSCs at P7. Left: Representative images. Tailless and trailed arrows indicate migrasomes without or with HERVK-Env (G) or dsRNA (H), respectively. Scale bars, 20 μm and 4 μm (zoomed-in images). Right: Quantification of the number of migrasomes with HERVK-Env (G) or dsRNA (H) per cell. *n* = 150 cells. ***P < 0.001 (*t* test). (I, J) Immunofluorescence co-staining of HERVK-Env (I) or dsRNA (J) with probe WGA probe in WT hMSCs at P8 treated with H_2_O_2_. Left: Representative images. Tailless and trailed arrows indicate migrasomes without or with HERVK-Env (I) or dsRNA (J), respectively. Scale bars, 20 μm and 4 μm (zoomed-in images). Right: Quantification of the number of migrasomes with HERVK-Env (I) or dsRNA (J) per cell. *n* = 150 cells. ***P < 0.001 (*t* test). (K) Immunofluorescence co-staining of HERVK-Env with WGA probe in human fibroblasts at EP (P12) and LP (P22). Left: Representative images. Tailless and trailed arrows indicate migrasomes without or with HERVK-Env, respectively. Scale bars, 20 μm and 4 μm (zoomed-in images). Right: Quantification of the number of migrasomes with HERVK-Env per cell. *n* = 150 cells. ***P < 0.001 (*t* test).

Intrigued by the above findings, we next asked whether additional constituents reported as senescent hallmarks might be present within migrasomes. We previously reported that heightened activity of a recently integrated subfamily of HERVs [42, 43], specifically HERVK, coupled with the accumulation and secretion of HERVK particles, represented a feature of cellular senescence, contributing to the mediation of pro-inflammatory effects in the microenvironment [9, 10, 44]. Given this discovery, we here asked whether migrasomes contain and facilitate the transport of senescence-associated endogenous retroviruses. We first asked whether the envelope (Env) protein of HERVK is present in migrasomes of senescent cells. Indeed, we detected an elevated number of migrasomes containing the Env protein in RS hMSCs transduced with lentiviral vectors carrying TSPAN4-GFP (Fig. 3A). Similarly, the accumulation of double-stranded RNA (dsRNA) (which is primarily derived from endogenous retrovirus transcripts and is the genetic material for viral particle packaging [45, 46]) in migrasomes was observed in LP hMSCs (Fig. 3B). We also detected increased presence of migrasomes containing HERVK-Env and dsRNA in RS hMSCs using WGA probe (Fig. 3C and D). To verify that senescence-associated endogenous retroviruses were accumulated in migrasomes, we isolated crude migrasomes from EP and LP hMSCs, and employed Western blotting analysis to confirm that HERVK-Env protein levels are elevated in migrasomes derived from LP hMSCs (Fig. 3E). Consistently, TEM analysis showed the presence of retrovirus-like particles (RVLP) within migrasomes in LP hMSCs (Fig. 3F), which was consistent with the RVLP observed in LP hMSCs (Supplementary Fig. S1I). Furthermore, we also observed increased enrichment of HERVK-Env and dsRNA within migrasomes in various senescent cell models, including pathological progeria (Fig. 3G and H), stress-induced prematurely senescent hMSCs (Fig. 3I and J), and RS fibroblasts (Fig. 3K). Taken together, these data indicate that endogenous retroviruses are present within migrasomes during cellular senescence.

### Pro-inflammatory cytokines were accumulated within migrasomes during cellular senescence

**Figure 4.**
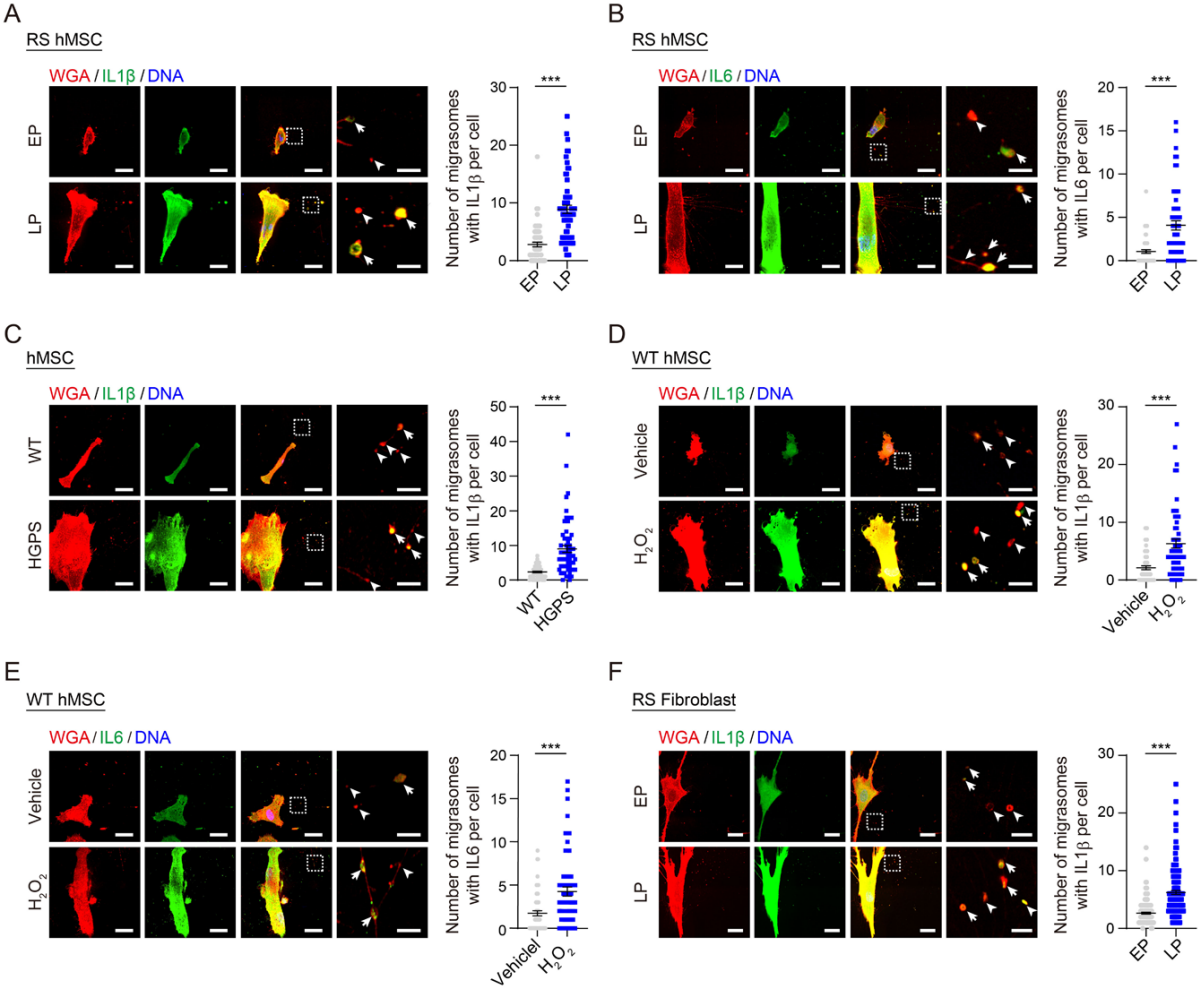
**Pro-inflammatory cytokines in migrasomes were enriched in senescent cells.** (A, B) Immunofluorescence co-staining of IL1β (A) or IL6 (B) with WGA probe in WT hMSCs at EP (P4) and LP (P14). Left: Representative images with long exposures. Tailless and trailed arrows indicate migrasomes without or with IL1β (A) or IL6 (B), respectively. Scale bars, 20 μm and 4 μm (zoomed-in images). Right: Quantification of the number of migrasomes with IL1β (A) or IL6 (B) per cell. *n* = 60 cells. ***P < 0.001 (*t* test). (C) Immunofluorescence co-staining of IL1β with WGA probe in WT and HGPS hMSCs at P7. Left: Representative images with long exposures. Tailless and trailed arrows indicate migrasomes without or with IL1β, respectively. Scale bars, 20 μm and 4 μm (zoomed-in images). Right: Quantification of the number of migrasomes with IL1β per cell. *n* = 60 cells. ***P < 0.001 (*t* test). (D-E) Immunofluorescence co-staining of IL1β (D) or IL6 (E) with WGA probe in WT hMSCs at P8 treated with H_2_O_2_. Left: Representative images with long exposures. Tailless and trailed arrows indicate migrasomes without or with IL1β (D) or IL6 (E), respectively. Scale bars, 20 μm and 4 μm (zoomed-in images). Right: Quantification of the number of migrasomes with IL1β (A) or IL6 (B) per cell. *n* = 60 cells. ***P < 0.001 (*t* test). (F) Immunofluorescence co-staining of IL1β with WGA probe in human fibroblasts at EP (P12) and LP (P22). Left: Representative images with long exposures. Tailless and trailed arrows indicate migrasomes without or with IL1β, respectively. Scale bars, 20 μm and 4 μm (zoomed-in images). Right: Quantification of the number of migrasomes with IL1β per cell. *n* = 150 cells. ***P < 0.001 (*t* test).

Resurrection of endogenous retroviruses during aging triggers activation of the innate immune response, which leads to increased expression of pro-inflammatory cytokines, known as SASP [9, 47–49]. Here, we asked whether migrasomes from RS hMSCs contain such senescence-associated inflammatory cytokines, in particular, Interleukin (IL)1β and IL6 [50, 51]. When we co-stained these cytokines with the WGA probe, we detected an increased number of migrasomes per cell that contained IL1β or IL6 in LP hMSCs relative to EP hMSCs (Fig. 4A and B). Of note, images were taken with long exposures to capture signals in the migrasomes. Furthermore, we also observed a heightened IL1β and IL6 enrichment in migrasomes from other senescent cell models (Fig. 4C–F), providing evidence for a potential role of migrasomes in the delivery of senescence-associated cytokines.

### Migrasomes mediated the transfer of aging signals in a paracrine manner

**Figure 5.**
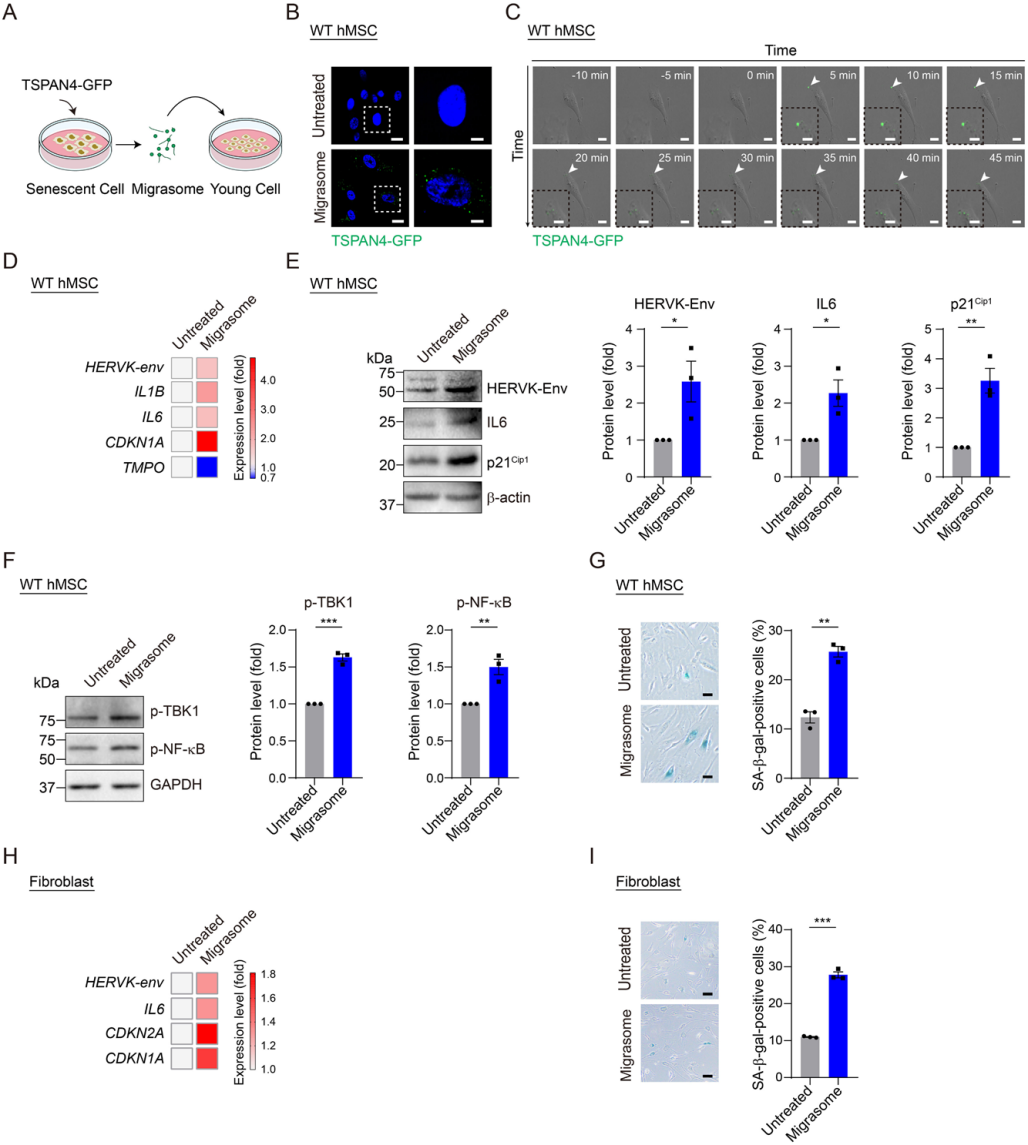
**Migrasomes derived from senescent cells accelerated senescence of treated cells.** (A) Schematic diagram illustrating the procedure of cell treatment with purified migrasomes from senescent hMSCs transduced with lentiviral vectors carrying TSPAN4-GFP. (B) Fluorescence microscopy detecting the GFP signals in EP (P6) WT hMSCs either untreated or treated with migrasomes purified from senescent hMSCs transduced with lentiviral vectors carrying TSPAN4-GFP. Scale bars, 30 μm. (C) Live-cell imaging recording the uptake of GFP-labeled migrasomes (white arrow) purified from senescent hMSCs transduced with lentiviral vectors carrying TSPAN4-GFP. The time adding migrasomes is marked as 0 min. The square frames in left bottom represent magnified images of the area indicated by white arrows. Scale bars, 20 μm and 40 μm (zoomed-in images). (D) Heatmap showing the expression level of HERVK-*env*, *IL1B* and *IL6*, as well as senescence-associated genes *CDKN1A* and *TMPO* in EP (P6) WT hMSCs either untreated or treated with migrasomes purified from senescent hMSCs transduced with lentiviral vectors carrying TSPAN4-GFP via qRT-PCR. β-actin was used as an internal control. (E) Western blotting of HERVK-Env, IL6 and p21^Cip1^ in EP (P6) WT hMSCs either untreated or treated with migrasomes purified from senescent hMSCs transduced with lentiviral vectors carrying TSPAN4-GFP. Left: Representative images. β-actin was used as loading control. Right: Quantification of the relative protein levels of HERVK-Env, IL6 and p21^Cip1^. Data are presented as the mean ± SEM. *n* = 3 independent experiments. *P < 0.05, **P < 0.01 (*t* test). (F) Western blotting of p-TBK1 and p-NF-κB in EP (P6) WT hMSCs either untreated or treated with migrasomes purified from senescent hMSCs transduced with lentiviral vectors carrying TSPAN4-GFP. Left: Representative images. GAPDH was used as loading control. Right: Quantification of the relative protein levels of p-TBK1 and p-NF-κB. Data are presented as the mean ± SEM. *n* = 3 independent experiments. *P < 0.05 (*t* test). (G) SA-β-gal staining of EP (P6) WT hMSCs either untreated or treated with migrasomes purified from senescent hMSCs transduced with lentiviral vectors carrying TSPAN4-GFP. Left: Representative images. Scale bars, 20 μm. Right: Quantification of the percentages of SA-β-gal-positive cells. Data are presented as the mean ± SEM. *n* = 3 biological replicates. Over 100 cells were quantified in each replicate. **P < 0.001 (*t* test). (H) Heatmap showing the expression level of *IL6*, and senescence-associated genes *CDKN1A* and *CDKN1A* in EP (P12) fibroblasts either untreated or treated with migrasomes purified from senescent fibroblasts transduced with lentiviral vectors carrying TSPAN4-GFP via qRT-PCR. β-actin was used as an internal control. (I) SA-β-gal staining of EP (P12) fibroblasts either untreated or treated with migrasomes purified from senescent hMSCs transduced with lentiviral vectors carrying TSPAN4-GFP. Left: Representative images. Scale bars, 20 μm. Right: Quantification of the percentages of SA-β-gal-positive cells. Data are presented as the mean ± SEM. *n* = 3 biological replicates. Over 100 cells were quantified in each replicate. ***P < 0.001 (*t* test).

Finally, we asked whether migrasomes released by senescent cells could be internalized by young cells, thereby amplifying pro-aging signals. To answer this question, we treated EP WT hMSCs with TSPAN4-GFP-labeled migrasomes purified from senescent hMSCs (Fig. 5A). The GFP signal was detected in young cells, indicating uptake of migrasomes by young cells (Fig. 5B). In addition, we also utilized live-cell imaging to observe the uptake of migrasomes by young cells (Fig. 5C and Supplementary Fig. S2A, Supplementary Movies S3 and S4). Consequently, we observed an increase in the levels of migrasome contents, which included HERVK-Env and IL6, in recipient cells, indicating that the migrasomes originating from senescent cells may transmit their pro-aging components into young hMSCs (Fig. 5D and E). Following migrasome treatment, we also noted an increase in the phosphorylation of TANK-binding kinase 1 (TBK1) and nuclear factor-κB (NF-κB), both prominent effectors in innate immune response [52], without a corresponding increase in their basal levels (Fig. 5F and Supplementary Fig. S2B). These data are consistent with our previous study in which we reported that activated endogenous retroviruses were released extracellularly and triggered innate immune response via the cyclic GMP-AMP synthase (cGAS)-stimulator of interferon genes (STING) pathway in recipient cells [9, 53]. Furthermore, these young cells displayed an accelerated senescence phenotype when treated with migrasomes derived from senescent cells compared to either untreated or treated with migrasomes from EP hMSCs as control (Fig. 5D, E, G and Supplementary Fig. S2C–E). This was demonstrated by an increased percentage of SA-β-gal-positive cells, elevated levels of senescence-associated gene *CDKN1A* (p21^Cip1^) [54, 55] and decreased expression level of *TMPO* (LAP2β) [54, 56] (Fig. 5D, E, G and Supplementary Fig. S2C–E). In addition, assays in fibroblasts also revealed an accelerated senescence phenotype in young fibroblasts after treatment with migrasome from senescent cells (Fig. 5H and I). Based on these data, we conclude that the accelerated senescence phenotypes in young cells may partially attribute to the activation of innate immunity-mediated inflammation triggered by components within migrasomes. Cumulatively, these findings suggest that migrasomes play a crucial role in delivering pro-aging signals.

## Discussion

**Figure 6.**
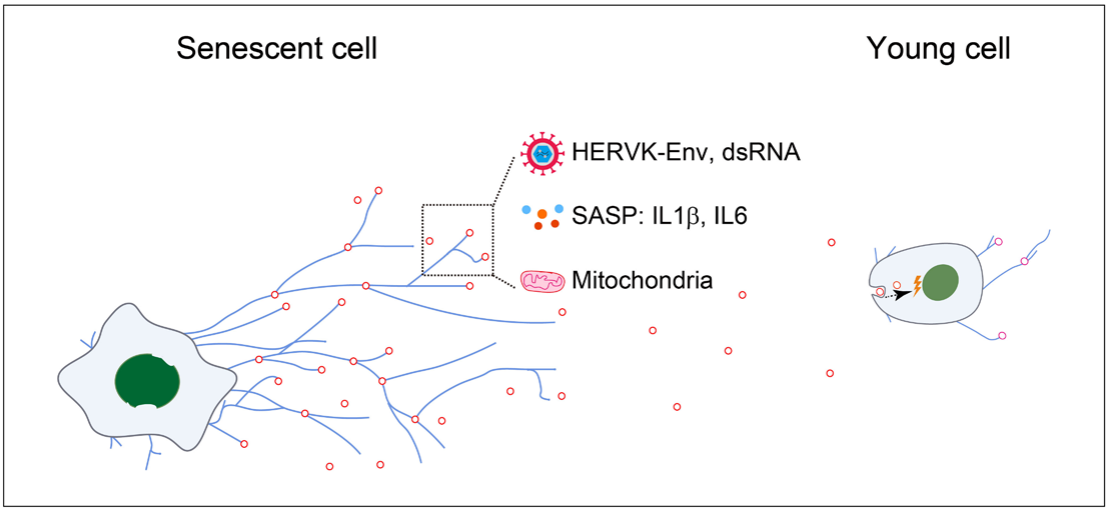
**Graphic summary.** Working model illustrates the important roles of migrasomes in delivery aging-associated biomarkers and transmission of pro-aging effect. The increased formation of migrasomes were observed in senescent cells, which are enriched with aging-associated biomarkers, including HERVK-Env, dsRNA, mitochondria, and inflammatory cytokines. The migrasomes released from senescent cells can be taken up by recipient young cells, thereby leading to their senescence.

In this study, we leveraged multiple human cellular senescence models to validate the formation and deposition of migrasomes as a novel mechanism associated with cellular senescence. We found that the number of migrasomes increased in senescent cells and that such migrasomes were enriched with molecules associated with aging-related hallmarks, including mitochondria, endogenous retroviruses and pro-inflammatory cytokines. Moreover, uptake of migrasomes derived from senescent cells caused activation of innate immune response in non-senescent cells, and thereby accelerated senescence in the recipient cells. In aggregate, these findings demonstrate that migrasomes are capable of delivering senescence-associated signals that transmit pro-aging effects (Fig. 6).

For the first time, we conducted a systemic investigation into the formation of migrasomes in multiple cellular senescence models, especially human mesenchymal stem cells, which have been widely applied in life science research given their self-renewal and multi-differentiation capabilities [57, 58]. Moreover, previous studies demonstrated that migrasomes act as chemoattractants by enriching CXCL12, thereby coordinating spatial organ morphogenesis during zebrafish gastrulation [21], and that migrasomes enriched with VEGFA and CXCL12 in monocytes promote angiogenesis during embryonic development in chickens [20]. Whether migrasomes also contribute to regulating tissue degeneration and organismal aging and can be transported to proximal or distal tissues through systemic circulation during aging *in vivo* requires further investigation.

As known that a variety of cytosolic agents are enriched in migrasomes [18, 19], we found that the content of migrasomes was highly heterogeneous. In migrasomes within the extracellular milieu of senescent cells, mitochondria were increased, in line with the notion that mitochondrial dysfunction is associated with aging [37–39]. Jiao *et al*. proposed that mitocytosis, a process in which migrasomes transport and depose damaged mitochondria upon exposure to mitochondrial stress, plays an important role in maintaining mitochondrial quality and homeostasis [36]. Therefore, the features and biological functions of depositing mitochondria into migrasomes during cellular senescence warrants further in-depth investigations. In this study, we found significantly increased levels of the endogenous viral protein and particles, and even the genetic material of viral dsRNA, in migrasomes of senescent cells. Of note, the origin of dsRNA in such migrasomes may also derive from other retroelements. These findings further prove our previous study that endogenous retroviruses act as a both biomarker and driver of aging. In addition, we observed inflammatory cytokines were also inside the migrasomes, which may stabilize cytokines released by senescent cells via protecting them from environmental degradation, thereby facilitating delivery of pro-aging signals to adjacent target cells. Further studies using multi-omics analysis are needed to comprehensively identify more senescence-associated biomarkers in migrasomes.

Importantly, we demonstrate that migrasomes play a functional role in delivering senescence-associated signals and transmitting pro-aging effects. This role entails the localized and systemic dissemination of aging signals between cells, in support of the concept of aging as a contagious process [11]. In fact, factors secreted by senescent cells (including inflammatory cytokines) have been known to induce senescence in nearby cells by other studies [11, 59], as well as our recent investigation showing that endogenous retroviruses released from senescent cells accelerated senescence of the receipt cells [9]. Here, we found that migrasomes derived from senescent cells can be taken up by young cells, contributing to the propagation of aging signals, thereby leading to an accelerated senescence of the recipient cells, partially through activation of innate immune response. Our findings demonstrate that migrasomes can be released in a paracrine manner and trigger cellular senescence in non-senescent cells, offering valuable insights into the concept of contagious aging and expanding our understanding of the mechanisms underlying the aging process. Moreover, in addition to cell movement [17], future investigations are warranted to determine whether and how other factors crucial for migrasome formation and maturation, such as integrins [12, 60], TSPAN4 [ [15, 61]], sphingomyelin synthase 2 (SMS2) [62], and the phosphatidylinositol (4, 5)-bisphosphate-Rab35 axis [63], are involved during aging.

Taken together, our study demonstrates that an increased number of migrasomes is a novel biomarker of aging, opening up new directions for further investigating the role of migrasomes in aging and providing novel intervention targets for mitigating aging and age-related diseases.

## Research limitations

We here demonstrated an increased number of migrasomes containing several aging biomarkers, including mitochondria, endogenous retroviruses, and pro-inflammatory cytokines, such as IL1β and IL6, in multiple senescent cell models. Further studies are needed to identify more senescence-associated biomarkers in migrasomes using multi-omics analysis. The mechanism underlying the formation and release of migrasomes by senescent cells also requires further investigation. Finally, the ability to use the understanding of migrasomes to achieve the regulation of aging represents a very interesting scientific question.

## Methods

### Cell culture and treatment

The culture condition for hMSCs followed established protocols [9, 26]. Briefly, hMSCs were cultured in hMSC culture medium consisting of 90% MEMα (Thermo Fisher Scientific), 10% fetal bovine serum (FBS, Thermo Fisher Scientific), 2 mM GlutaMAX (Thermo Fisher Scientific), 0.1 mM non-essential amino acids (NEAA, Thermo Fisher Scientific), 1% penicillin/streptomycin (Thermo Fisher Scientific), and 1 ng/ml bFGF (Joint Protein Central). The cells were grown on 0.1% gelatin (Sigma Aldrich)-coated plates (CORNING). To establish stress-induced senescence model, EP WT hMSCs were treated with 100 μM H_2_O_2_ (Sigma Aldrich) for 10 h [33]. For migrasome treatment, EP WT hMSCs were seeded and incubated with purified migrasomes from LP WT hMSCs transduced with lentiviral vectors carrying TSPAN4-GFP for two passages. The medium supplemented with migrasomes was changed every 2 days. Dulbecco’s Modified Eagle Medium (Thermo Fisher Scientific) was used to culture human fibroblasts and HEK293T cells supplemented with 10% FBS, 2 mM GlutaMAX, 1% penicillin/streptomycin, and 0.1 mM NEAA. All cells were maintained at 37°C in a humidified incubator (Thermo Fisher Scientific) with 5% CO_2_.

### Crude migrasome purification

To purify migrasomes, cells seeded on gelatin-coated dishes were trypsinized and collected after washing with phosphate-buffered saline (PBS). The purification of migrasomes were conducted according to previous studies [18, 20]. In brief, the samples were centrifuged at 1000 g for 10 min to eliminate cell bodies. The supernatants were collected and further centrifuged at 4000 g for 20 min to remove cell debris. The crude migrasomes were collected by subjecting the samples to ultracentrifugation at 4°C at a speed of 20,000 g for 40 min followed by washing with PBS. Then, the resulting pellets were resuspended in PBS and subsequently used for western blotting and negative-staining TEM analysis, as well as cell treatment.

### Western blotting

Whole-cell or purified migrasomes were lysed in 2% SDS buffer containing 200 mM Tris–HCl (pH = 6.8), 20% glycerol, 1% SDS, and 4% 2-mercaptoethanol. After measuring the protein concentration using the BCA Kit (Dingguochangsheng Biotech), the same amount of protein lysates was boiled at 105°C for 10 min and resolved on an SDS-PAGE gel. The separated proteins were transferred onto PVDF membranes (Millipore). Subsequently, the blots were blocked with 5% non-fat milk (Sangon Biotech) and incubated with the primary antibodies at 4°C overnight and HRP-conjugated secondary antibodies at room temperature for 1 h. After incubation with substrates, the blots were imaged using a ChemiDoc XRS + system (Bio-Rad Laboratories, Inc.) with an Image Lab software. ImageJ was used to quantify the protein intensity.

### Lentiviral transduction of TSPAN4-GFP construct

The human TSPAN4-GFP cDNA was amplified and subcloned to the pLE4 vector, generously provided by Tomoaki Hishida. Lentivirus package and transduction followed established methods [9, 37]. Briefly, pLE4-TSPAN4-GFP were cotransfected with the packaging plasmids pMD2.G (Addgene, #12260) and psPAX2 (Addgene, #12259) into HEK293T using Lipofectamine 3000 (Thermo Fisher Scientific). The culture medium was collected, and lentivirus particles were concentrated through ultracentrifugation at 19,400 r/min for 2.5 h at 4°C. The same amount of TSPAN4-GFP lentivirus was then added to the EP and LP hMSCs in the presence of polybrene (Sigma-Aldrich) for 24 h. After passaging, the transfected cells were used for immunofluorescence staining, live-cell imaging, and migrasome purification.

### RNA isolation and quantitative reverse-transcription PCR (qRT-PCR)

Following treatment with purified TSPAN4-GFP-labeled migrasomes, the total RNA was isolated from the cells using TRIzol (Thermo Fisher Scientific). GoScript Reverse Transcription System (Promega) was used for reverse transcription to synthesize the first strand cDNA. The expression levels of indicated genes were determined via qPCR using the THUNDERBIRD qPCR Mix (TOYOBO) on a CFX384 Real-Time System (Bio-Rad Laboratories, Inc.). The primer sequences used are based on previously published work.

### Immunofluorescence staining and microscopy

The procedure of immunofluorescence staining of migrasomes were followed by previous studies [12, 15]. Briefly, cells were seeded on gelatin-coated coverslips (Thermo Fisher Scientific) overnight and fixed with 2.5% glutaraldehyde for 10 min followed by permeabilization in 0.4% Triton X-100 for 10 min and blocking with 10% donkey serum for 1 h. The cells were then incubated with indicated primary antibodies at 4°C overnight and then secondary antibodies or Alexa Fluor™ 555-conjugated WGA probe (Invitrogen) at room temperature for 1 h. Nuclei were labeled with Hoechst 33342 (Thermo Fisher Scientific). In order to faithfully detect the intact structure of the migrasome, all operations were carefully performed using gentle techniques and without any shaking. The images of migrasomes were captured with long exposures by a Dragonfly confocal microscopy system (Andor).

### Live-cell imaging

For live-cell imaging, the TSPAN4-GFP transfected hMSCs were seeded at a density of 4 × 10^4^ per well in confocal dishes (Nest, 801001). After 24 h, live-cell imaging was carried out at indicated time points using a Dragonfly 488 fluorescence confocal microscopy (Andor) with a temperature-controlled heating system. The cells were imaged with a 64 × oil objective lens, and images were captured every 5 min for 6.5 h period.

### Antibodies

The following antibodies were used for western blotting and immunofluorescence staining: anti-HERVK-Env (Austral Biologicals), anti-dsRNA (Kerafast), anti-IL1β (Santa Cruz Biotechnology), anti-IL6 (Peprotech), Tom20 (Santa Cruz Biotechnology), anti-p-NF-κB (Cell Signaling), anti-p-TBK1 (Cell Signaling), anti-NF-κB (Cell Signaling), anti-TBK1 (Cell Signaling), anti-GFP (Santa Cruz Biotechnology), anti-β-actin (Santa Cruz Biotechnology), anti-GAPDH (Santa Cruz Biotechnology), Alexa Fluo^TM^ 488 Donkey Anti-Rabbit IgG (H+L) (Invitrogen), Alexa Fluo^TM^ 488 Donkey Anti-Mouse IgG (H+L) (Invitrogen), HRP-conjugated Goat Anti-Mouse IgG (H+L) (ZSGB-Bio), and HRP-conjugated Goat Anti-Rabbit IgG (H+L) (ZSGB-Bio).

### SA-β-gal staining

SA-β-gal staining was conducted following established protocols [9, 37]. Cells were seeded on gelatin-coated plates, followed by fixation in 2% (w/v) formaldehyde and 0.2% (w/v) glutaraldehyde for 5 min and staining with X-gal staining buffer at 37°C overnight. The SA-β-gal-positive cells were visualized and captured by an optical microscope and calculated using ImageJ software.

### Clonal expansion assay

Clonal expansion assay was as performed previously described [9, 37]. Five thousand cells were seeded and grown for approximately 10 days in gelatin-coated six-well plate (Corning). After fixation with 4% PFA for 30 min, the cells were stained with 10% crystal violet for 30 min. ImageJ software was utilized to determine the relative cell density.

### Transmission electron microscopy

The procedure of transmission electron microscopy was conducted according to previous publications [14, 36]. Briefly, cells were grown on gelatin-coated 35-mm Petri dishes (CORNING) overnight and prefixed with warmed 2.5% glutaraldehyde and culture medium mixture at a ratio of 1:1 at 37°C in a cell incubator for 5 min. Cells were then further fixed with 2.5% glutaraldehyde at room temperature for 1 h and 4°C overnight. After dehydration in an ascending gradual series of ethanol (50%, 70%, 90%, 95%, and 100%) for 8 min each, samples were infiltrated and embedded in SPON12 resin and polymerized for 48 h at 60°C. A diamond knife was used to the samples into 70-nm-thick ultrathin sections. Then the sections were picked up with Formvar-coated copper grids (100 mesh) and double stained with uranyl acetate and lead citrate. After air drying, a TEM Spirit 120 kV (FEI Tecnai Spirit 120 kV) was used for imaging. For negative-staining TEM analysis, the purified migrasomes were deposited onto copper mesh with ultra-carbon foil grids, which had a mesh size of 230. Subsequently, the grids were stained with 2% uranyl acetate. After staining, the grids were observed using a transmission electron microscope (ThermoFisher Tecnai Spirit, 120 kV) equipped with an EMSIS Veleta camera (2K × 2K).

### Research ethics

The WT and HGPS hMSCs derived from human embryonic stem cell H9 (WiCell Research Institute) via direct *in vitro* differentiation and gene editing established by previous study [26]. The primary fibroblasts used in this study were the same ones reported in the previous studies [64]. There are no relevant ethical issues.

### Statistical analysis

The Prism version 8 software (GraphPad Software) was used for statistical analyses. The data were presented as means ± SEM. Comparisons were made by the two-tailed Student's *t* test. Statistical significance was denoted as follows: ns (not significant), * for *P* < 0.05 (statistically significant), ** for *P* < 0.01 (highly statistically significant), and *** for *P* < 0.001 (highly statistically significant).

## Data availability

The data supporting the findings of this study are available within the article and its supplementary materials.

## Supplementary Material

lnad050_suppl_Supplementary_Figures_S1

lnad050_suppl_Supplementary_Figures_S2

lnad050_suppl_Supplementary_Movies_S1

lnad050_suppl_Supplementary_Movies_S2

lnad050_suppl_Supplementary_Movies_S3

lnad050_suppl_Supplementary_Movies_S4

lnad050_suppl_Supplementary_Data
