# Supplementary figures and images for: Migrasomes trigger innate immune activation and mediate transmission of senescence signals across human cells
Source: Life Med. 2023 Dec 8;2(6):lnad050. doi: 10.1093/lifemedi/lnad050 (PMC11749555; doi:10.1093/lifemedi/lnad050)

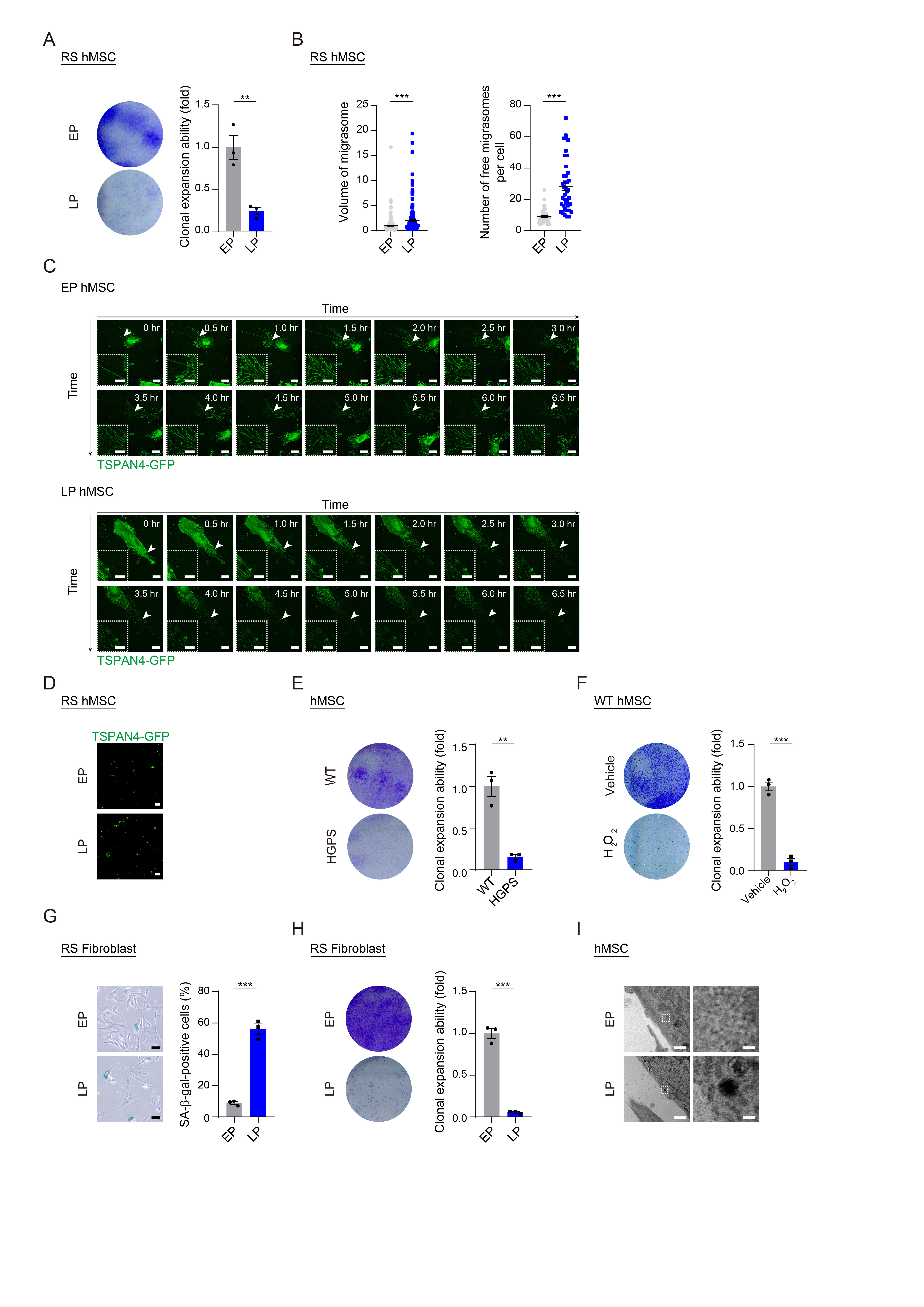

Supplement: lnad050_suppl_Supplementary_Figures_S1 [file lnad050_suppl_Supplementary_Figures_S1.jpg]

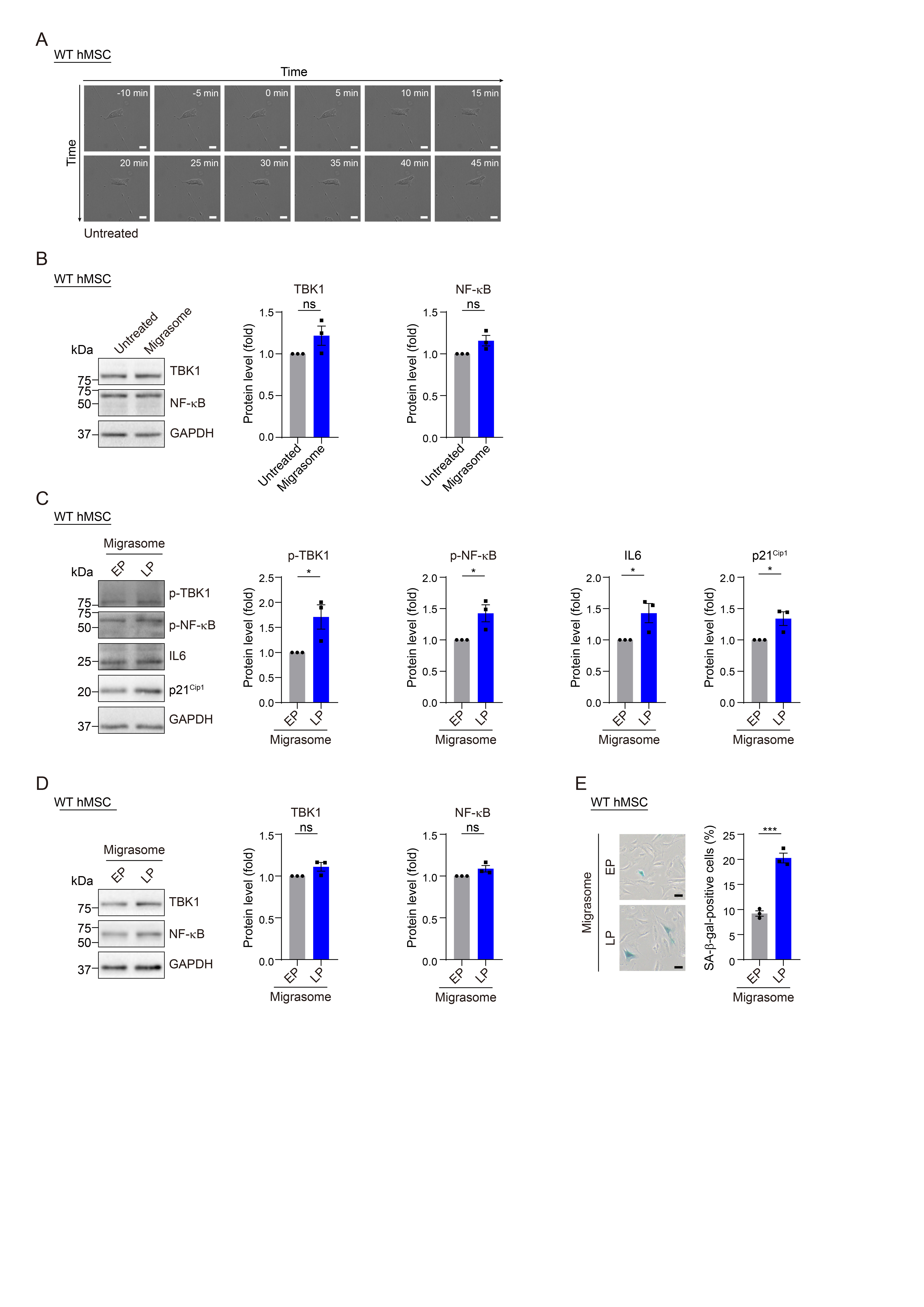

Supplement: lnad050_suppl_Supplementary_Figures_S2 [file lnad050_suppl_Supplementary_Figures_S2.jpg]
