# Supplementary material for: Migrasomes trigger innate immune activation and mediate transmission of senescence signals across human cells
Source: Life Med. 2023 Dec 8;2(6):lnad050. doi: 10.1093/lifemedi/lnad050 (PMC11749555; doi:10.1093/lifemedi/lnad050)
Supplement: lnad050_suppl_Supplementary_Data [file lnad050_suppl_Supplementary_Data.docx]

**Supplementary information**

**Migrasomes trigger innate immune activation and mediate transmission of senescence signals across human cells**

Xiaoqian Liu^1,2,3,4,‡^, Haifeng Jiao^5,‡^, Baohu Zhang^1,2,‡^, Sheng Zhang^2,6^, Kaowen Yan^2,3,4,6^, Jing Qu^1,2,3,4,7,*^, Weiqi Zhang^2,3,7,8,*^, Li Yu^5,*^, Guang-Hui Liu^2,3,4,6,7,9,*^

^1^State Key Laboratory of Stem Cell and Reproductive Biology, Institute of Zoology, Chinese Academy of Sciences, Beijing 100101, China

^2^University of Chinese Academy of Sciences, Beijing 100049, China

^3^Institute for Stem Cell and Regeneration, Chinese Academy of Sciences, Beijing 100101, China

^4^Beijing Institute for Stem Cell and Regenerative Medicine, Beijing 100101, China

^5^State Key Laboratory of Membrane Biology, Tsinghua University-Peking University Joint Center for Life Sciences, Beijing Frontier Research Center for Biological Structure, School of Life Sciences, Tsinghua University, Beijing 100084, China.

^6^State Key Laboratory of Membrane Biology, Institute of Zoology, Chinese Academy of Sciences, Beijing 100101, China

^7^Aging Biomarker Consortium, Beijing 100101, China

^8^CAS Key Laboratory of Genomic and Precision Medicine, Beijing Institute of Genomics, Chinese Academy of Sciences and China National Center for Bioinformation, Beijing 100101, China

^9^Advanced Innovation Center for Human Brain Protection, and National Clinical Research Center for Geriatric Disorders, Xuanwu Hospital Capital Medical University, Beijing 100053, China

‡These authors contributed equally to this work.

*Correspondence: qujing@ioz.ac.cn (J.Q.), zhangwq@big.ac.cn (W.Z.), [liyulab@mail.tsinghua.edu.cn](mailto:liyulab@mail.tsinghua.edu.cn) (L.Y.), ghliu@ioz.ac.cn (G.-H.L.)

**Figure S1. Establishment of senescence cell models.**

(A) Clonal expansion assay in WT hMSCs at EP (P4) and LP (P14) WT hMSCs. Left: Representative images. Right: Quantification of the relative clonal expansion ability. Data are presented as the mean ± SEM. *n* = 3 biological replicates. ** *P* < 0.01, *** *P* < 0.001 (*t* test). (B) Quantification of the volume of migrasomes and the number of released migrasomes per cell in Fig. 1C. *** *P* < 0.001 (*t* test). (C) Live-cell imaging recording the formation and movement of migrasomes in EP (P4) and LP (P14) WT hMSCs transduced with lentiviral vectors carrying TSPAN4-GFP. The square frames in left bottom represent magnified images of the area indicated by white arrows. Scale bars, 20 μm and 40 μm (zoomed-in images). (D) Fluorescence microscopy analysis of purified migrasomes from EP (P4) and LP (P14) WT hMSCs transduced with lentiviral vectors carrying TSPAN4-GFP. Scale bars, 2 μm. (E, F) Clonal expansion assay in WT and HGPS hMSCs at P7 (C), or in WT hMSCs at P8 treated with H_2_O_2_ (D). Left: Representative images. Right: Quantification of the relative clonal expansion ability. Data are presented as the mean ± SEM. *n* = 3 biological replicates. ** *P* < 0.01, *** *P* < 0.001 (*t* test). (G, H) SA-β-gal staining (E) or clonal expansion assay (F) of human fibroblasts at EP (P12) and LP (P22). Left: Representative images. Scale bars, 20 μm. Right: Quantification of the percentages of SA-β-gal-positive cells (E) and the relative clonal expansion ability (F). Data are presented as the mean ± SEM. *n* = 3 biological replicates. Over 100 cells were quantified in each replicate for (E). *** *P* < 0.001 (*t* test). (I) TEM analysis of RVKPs within migrasomes in WT hMSCs at EP (P4) and LP (P14). Scale bars, 1 μm and 100 nm (zoomed-in images).

**Figure S2. Treatment with migrasomes derived from senescent cells accelerated senescence of the recipient cells.**

(A) Live-cell imaging recording the untreated cells in Fig. 5C. The time adding migrasomes is marked as 0 min. Scale bars, 20 μm. (B) Western blotting of TBK1 and NF-κB in EP (P6) WT hMSCs either untreated or treated with migrasomes purified from senescent hMSCs transduced with lentiviral vectors carrying TSPAN4-GFP. Left: Representative images. GAPDH was used as loading control. Right: Quantification of the relative protein levels of TBK1 and NF-κB. Data are presented as the mean ± SEM. *n* = 3 independent experiments. ns, not significant (*t* test). (C) Western blotting of IL6, p21^Cip1^, p-TBK1 and p-NF-κB in EP (P6) WT hMSCs treated with migrasomes purified from either young or senescent hMSCs transduced with lentiviral vectors carrying TSPAN4-GFP. Left: Representative images. GAPDH was used as loading control. Right: Quantification of the relative protein levels of IL6, p21^Cip1^, p-TBK1 and p-NF-κB. Data are presented as the mean ± SEM. *n* = 3 independent experiments. * *P* < 0.05 (t test). (D) Western blotting of TBK1 and NF-κB in EP (P6) WT hMSCs treated with migrasomes purified from either young or senescent hMSCs transduced with lentiviral vectors carrying TSPAN4-GFP. Left: Representative images. GAPDH was used as loading control. Right: Quantification of the relative protein levels of TBK1 and NF-κB. Data are presented as the mean ± SEM. *n* = 3 independent experiments. ns, not significant (*t* test). (E) SA-β-gal staining of EP (P6) WT hMSCs treated with migrasomes purified from either young or senescent hMSCs transduced with lentiviral vectors carrying TSPAN4-GFP. Left: Representative images. Scale bars, 20 μm. Right: Quantification of the percentages of SA-β-gal-positive cells. Data are presented as the mean ± SEM. *n* = 3 biological replicates. Over 100 cells were quantified in each replicate. *** *P* < 0.001 (*t* test).

Movie S1: Live-cell imaging of EP (P4) WT hMSCs transduced with lentiviral vectors carrying TSPAN4-GFP.

Movie S2: Live-cell imaging of LP (P14) WT hMSCs transduced with lentiviral vectors carrying TSPAN4-GFP.

Movie S3: Live-cell imaging recording the untreated cells.

Movie S4: Live-cell imaging recording the uptake of GFP labeled migrasomes purified from senescent hMSCs transduced with lentiviral vectors carrying TSPAN4-GFP by treated recipient cells.
